# *In Vitro* Infection with Dengue Virus Induces Changes in the Structure and Function of the Mouse Brain Endothelium

**DOI:** 10.1371/journal.pone.0157786

**Published:** 2016-06-23

**Authors:** Myriam L. Velandia-Romero, María-Angélica Calderón-Peláez, Jaime E. Castellanos

**Affiliations:** Laboratorio de Virología, Universidad El Bosque, Bogotá, Colombia; Instituto Nacional de Salud Pública, MEXICO

## Abstract

**Background:**

The neurological manifestations of dengue disease are occurring with greater frequency, and currently, no information is available regarding the reasons for this phenomenon. Some viruses infect and/or alter the function of endothelial organs, which results in changes in cellular function, including permeability of the blood-brain barrier (BBB), which allows the entry of infected cells or free viral particles into the nervous system.

**Methods:**

In the present study, we standardized two in vitro models, a polarized monolayer of mouse brain endothelial cells (MBECs) and an organized co-culture containing MBECs and astrocytes. Using these cell models, we assessed whether DENV-4 or the neuro-adapted dengue virus (D4MB-6) variant infects cells or induces changes in the structure or function of the endothelial barrier.

**Results:**

The results showed that MBECs, but not astrocytes, were susceptible to infection with both viruses, although the percentage of infected cells was higher when the neuro-adapted virus variant was used. In both culture systems, DENV infection changed the localization of the tight junction proteins Zonula occludens (ZO-1) and Claudin-1 (Cln1), and this process was associated with a decrease in transendothelial resistance, an increase in macromolecule permeability and an increase in the paracellular passing of free virus particles. MBEC infection led to transcriptional up-regulation of adhesion molecules (VCAM-1 and PECAM) and immune mediators (MCP-1 and TNF- α) that are associated with immune cell transmigration, mainly in D4MB-6-infected cells.

**Conclusion:**

These results indicate that DENV infection in MBECs altered the structure and function of the BBB and activated the endothelium, affecting its transcellular and paracellular permeability and favoring the passage of viruses and the transmigration of immune cells. This phenomenon can be harnessed for neurotropic and neurovirulent strains to infect and induce alterations in the CNS.

## Introduction

The neurovascular unit (NVU) of the blood-brain barrier (BBB) consists of astrocytes, pericytes, neurons and endothelial cells, which control the selectively of the passage of molecules from capillaries into the brain parenchyma and vice versa [1, 2, 3]. This selectivity is altered when endothelial cells are infected with several types of viruses, such as HIV, rabies virus, herpes simplex virus, West Nile Virus (WNV) and Japanese encephalitis virus (JEV) [4]. Endothelial infection induces an activation status that affects its function and dramatically changes its barrier functions, mainly its permeability and selectivity [5, 6]. These changes facilitate plasma leakage and the entry of viruses or infected cells into the brain parenchyma, which promotes the spread of the virus [4].

In recent years, it has been shown that infection with dengue virus (DENV) can affect organs, including the liver, heart, kidneys, and brain [5]. In dengue cases where there are neurological symptoms, it has been reported that motor, sensory and cognitive alterations; encephalitis; encephalomyelitis; transverse myelitis; behavioral disorders; flaccid paralysis; and Guillain-Barré syndrome can occur because of the replication of the virus or local or systemic immune responses to infection [7, 8, 9]. It is clear that DENV infection and the spread of the virus into different organ tissues depends on the infection and/or alterations in endothelial cells [5]. It has been demonstrated both in vitro and in necropsies that DENV can infect and replicate in endothelial cells in organs such as the liver, pleura, pericardium, lungs, urinary tract, intestines and brain [6, 10, 11], thereby allowing the passage of the virus from the lumen into the stromal tissues. In addition, establishment of a powerful immune response, including high serum levels of cytokines and chemokines, directly contributes to endothelial activation, which modifies tissue permeability and allows immune cell infiltration, plasma leakage and the imbalance of coagulation that is associated with dengue disease [5, 6, 12, 13, 14].

Recently, Hapuarachchi and colleagues reported a fatal case of DENV-4, in which rapid neurological deterioration was induced from the onset of the disease. Viral RNA was detected in both the serum and the cerebrospinal fluid, but the latter of these two showed a higher titer. This finding suggests that the rapid neurological deterioration observed in the patient was the result of early entry of the virus into the central nervous system (CNS) through the BBB [15]. Experimentally, it was shown that intracerebral inoculation of DENV-2 into adult mice altered the permeability of the BBB and stimulated the infiltration of immune cells and plasma proteins into the brain parenchyma, which altered neurological function [16]. Similarly, our group used a model of dengue neuro-infection in suckling mice and reported that the infection of neurons and microglia accompanied BBB alterations, shown mainly by the infiltration of immune cells and by Evans blue extravasation toward the brain parenchyma after extraneural inoculation with the neuro-adapted strain [9]. These results suggest that the infection and the local and systemic immune responses affected the BBB and promoted the development of neurological alterations following exposure to this DENV strain. In spite of such evidence, the roles of the cerebrovascular endothelium and BBB alterations in the entry and spread of the virus in nervous tissue and in the development of neurological signs associated with DENV have not been described.

It is likely that the infection and subsequent alterations to endothelial cells promote DENV entry into nervous tissues, leading to brain infection and neuropathogenesis. Therefore, in the present work, we standardized two *in vitro* BBB systems using brain endothelial cells with or without astrocytes that were obtained from neonatal mice to assess whether two DENV variants, DENV-4 or the neuro-adapted DENV (D4MB-6), could infect and induce changes in the function of the cerebrovascular endothelium to promote the passage of the virus from one side of the barrier to the other.

## Materials and Methods

### Animals and welfare considerations

All procedures described in the present study related to obtaining the cells and producing the neuro-adapted virus were approved by the Ethics Committee of the Universidad El Bosque (Bogotá, Colombia) in consideration of the international and Colombian regulations for handling animals. Mouse brain endothelial cells (MBECs) and astrocytes were obtained from 7-day-old postnatal Balb/C mice.

1. Housing and husbandry: The animals were kept in the animal facility (Universidad Nacional de Colombia), in a private room that had all the requirements for proper care of animals. In this, the mice were kept in home cages (according to the size of the animals), covered with sterile wood chip. The top of the cage was a metal net with a special place to put the food with all the nutritional requirements of the species and a water bottle. Each cage had a male and female mice (Balb/C) with their pups. All home cages were properly labeled and birth date and the number of offspring at each birth are recorded. During the experimental period the animals moved to clean cages with new food and water twice a week.
2. Animal physical condition monitoring: Every day, during experimentation period we observed and register the typical behavior of animals (maintenance, exploration, affiliate interactions, sexual and maternal behaviors). When we observed abnormal behavior, this was informed to veterinarian.
3. Steps to minimize pain and distress: During the experimental period only two sick animals were observed before the endpoint. Sick animals, were killed by cervical dislocation or overdose of anesthetic in a private room at the animal facility. The bodies were stored in the cold until incineration. An adult female had a tumor in peritoneum and the other one died from an unknown cause.
4. Protocol for the early euthanasia/humane endpoints for animals who became severely ill/moribund during the experiment(s): If the animals show some signs such as weight loss, poor appetite, impaired movement, visible tumors, or abnormal behavior for more than two days, we informed to veterinarian and they were immediately sacrificed (overdose of anesthetic or cervical dissociation) and the bodies were frozen until incineration. Harvesting of neuroadapted virus was made at 6 days post infection, before animals were moribund and they were sacrificed by cervical dislocation when they presented hind limbs paralysis.
5. Euthanasia method for all animals utilized in this research: Postnatal pups of seven days used for the cultures, were euthanized with an overdose of anesthetic (ketamine and Xilaxine).

### Viruses

The D4MB-6 viral variant was obtained following a published protocol [9]. Briefly, *Aedes albopictus* C6/36HT cells were inoculated with a DENV-4 serotype isolate, and the supernatants were harvested. This virus was inoculated three successive times into SH-SY5Y neuroblastoma cells (ATCC). The third passage was intracerebrally inoculated into Balb/C suckling mice, recovered six days later, and then re-inoculated five more times to obtain the dengue serotype 4 mouse brain passaged six times strain (neuro-adapted D4MB-6). Brains were dissected from anesthetized mice, homogenized in 10% tissue suspensions and titrated using a plaque assay into LLMCK2 (ATCC) cells (2.6 X 10^6^ PFU/ml). Non-infected brain lysate or C6/36HT supernatants were used as mock inocula.

### Culture, characterization and infection of astrocytes from neonatal mouse brains

Astrocytes were isolated according to a modified protocol from Skaper and colleagues [17]. Seven-day-old mice were sacrificed with an anesthetic overdose, and their brains were extracted to dissect the cortex. Tissue was digested in a collagenase (2 mg/ml, Gibco), dispase (2 mg/ml, MP Biomedicals), DNAse (0.4 mg/ml, MP Biomedicals), GlutaMAX (0.7 mM, Gibco) and L-cysteine (0.72 mg/ml, Merck) solution for 1 hour at 37°C. Then, the tissue suspension was homogenized in an ovomucoid solution (0.7 mM GlutaMAX, 0.4 mg/ml DNAse, 0.15 mg/ml of bovine serum albumin (BSA), and 3 mg/ml trypsin inhibitor) and settled for 2 min at 37°C. Then, the pellet was resuspended in a 1:3 mix of ovomucoid solution and maintenance medium (MM; DMEM plus 10% HyClone FBS and penicillin/streptomycin) until the tissue was completely dissociated. Finally, the cell suspension was centrifuged at 200 g, resuspended in MM and seeded in 75 cm^2^ flasks and maintained for 14 days. Subsequently, the culture was purified using agitation in a horizontal shaker for 16 h at 37°C, and the adherent cells (astrocytes) were maintained in MM for eight additional days.

The astrocytes were trypsin-detached, and 8,000 cells were seeded on 10 or 100 μg/ml poly-L-lysine-treated round glass coverslips or Transwell inserts (0.3 cm^2^, 3-μm pores). Twenty-four hours later, the cells were fixed with 4% paraformaldehyde (PFA), permeabilized with Triton-X100 and processed to detect glial fibrillary acidic protein (GFAP, Dako, Z03334) or glutamate transporter (GLT-1, SIGMA, SAB2102171). Then, the cells were incubated with a secondary biotinylated anti-rabbit antibody (Vector) and Alexa-488-coupled streptavidin (Invitrogen, S11223). To assess infection percentages, astrocytes cultured on glass coverslips were infected with the DENV-4 or D4MB-6 variants at a multiplicity of infection (MOI) of 1 for 1 h at 37°C. The viruses were removed, and the cells were then cultured for 24 or 48 additional hours. After this post-infection (p.i.) period, the cells were fixed with 4% paraformaldehyde (PFA) and processed for immunofluorescence to simultaneously detect the virus E protein using an anti-flavivirus antibody (Millipore, MAB 8744) and GFAP, as previously described. Finally, the coverslips or transwell membranes were mounted on glass slides using Vectashield and analyzed under a microscope (Zeiss AxioImager A2) using a fluorescence X-Cite series 120Q system and Axio Vision software.

### Culture, characterization and infection of mouse cerebrovascular endothelial cells (MBECs)

MBECs were isolated using the standardized protocol of Weidenfeller and colleagues [18], with some modifications. Briefly, the cerebral cortex was mechanically dissociated in a solution of DNAse (0.15 ng/ml) and collagenase (1 mg/ml) for 1 h at 37°C. The solution was then centrifuged at 300 g for 8 minutes, and the pellet was loaded in a solution of 20% BSA and centrifuged thrice at 1000 g for 5 minutes. The pellet was dissociated using a DNAse (0.06 ng/ml) and collagenase/dispase (1 mg/ml) solution for 50 min at 37°C in shaker, and the tissue suspensions were then centrifuged on a 33% Percoll gradient at 1000 g for 10 min. The resulting pellet contained the isolated microvessels, which were seeded in 12-well plates and maintained for three days in DMEM/F12 culture medium supplemented with puromycin (3 μg/ml) [19]. The cells were maintained for 30 days in DMEM/F12 (Sigma) with 20% FBS, penicillin/streptomycin, 0.7 mM GlutaMAX, 15 U/ml heparin, 1 ng/ml basic fibroblast growth factor (bFGF) and astrocyte conditioned medium. After reaching confluence, the cells were dissociated, and 12,000 MBECs were seeded on Transwell^®^ inserts (polyester membrane; Corning) or glass coverslips that were pretreated with 10 μg/ml of collagen type IV (Sigma) and fibronectin (Invitrogen). To detect ZO-1 (Invitrogen, 71–6300) or von Willebrand Factor (vWF, Abcam, ab6994), the MBECs were fixed with 4% PFA and processed using the protocol described above. To detect Claudin-1 –Cln1- (Santa Cruz, sc-17658), the cells were methanol-fixed, permeabilized with Triton X-100, and washed with Ca^++^/Mg^++^ PBS [20]. Then, the cells were incubated for 1 h at 37°C with the primary antibody, washed and incubated for 30 min with the secondary biotinylated antibody (anti-goat 1:200; Vector, BA-9500), and then incubated with different Alexa-coupled streptavidin solutions: Alexa-488 (Invitrogen, S11223), Alexa-594 (Invitrogen, S11227) or Alexa-647 (Invitrogen, S21374). To assess DENV infection, the MBECs that were seeded on glass coverslips were infected for 1 h at 37°C with DENV-4 or D4MB-6 viruses, as previously described, for 24 or 48 additional hours. Infected cultures were fixed and processed to detect the E protein of DENV (Millipore, MAB 8744) and ZO-1 and analyzed as previously described.

### Standardization of two barrier models: Monolayer (MBECs) and co-culture (MBECs-astrocytes)

To standardize both models, we used Transwell^®^ inserts (0.3 cm^2^ with 0.4 or 3 μm pores). For the monolayer model, 30,000 MBECs were seeded on the luminal side of the membrane and maintained in endothelial MM. Cell confluence was assessed daily by measuring transendothelial electrical resistance (TEER, measured using a voltmeter; Millicell-ERS^®^). System permeability was quantified by passing Blue Dextran through the membrane at different time points. For the co-culture model, the abluminal side of the membrane was first treated with poly-L-lysine (100 μg/ml), and then 30,000 astrocytes were seeded and allowed to adhere for 3 days at 37°C, according to the protocol of Zhang et al [21]. On the fourth day, the inserts were turned upside down, and 30,000 MBECs were seeded at the luminal side of the membrane, and TEER was evaluated. The changes in resistance and permeability that occurred after DENV infection were then evaluated. MBECs in both barrier systems were infected with DENV-4 or D4MB-6 virus, as previously described. Then, 2.4 mg/ml Blue Dextran 2000 (Fine Chemicals) plus 10% FBS were added to the culture medium [22] and changed every 2 h for the first 12 h p.i. and at 24 and 48 h p.i. Absorbance was read at 630 nm in lower chamber media, and TEER values were measured. Additionally, the upper and lower media were collected during each change for virus titration. Finally, the membranes were processed for fluorescence microscopy to detect viral and endothelial markers, as previously described.

### Quantification of transcripts

To quantify mRNA levels in MBECs (monolayer model), 200,000 cells were seeded on 1.2 cm^2^ Transwells^®^ (0.4 μm pore size), and the cells were maintained for 4 days. They were then infected at an MOI of 1 with DENV-4 or D4MB-6 for defined time periods. Total RNA was purified using a conventional TRIzol^®^ method, and 250 ng of RNA was retro-transcribed, amplified and quantified using an enzyme superscript^®^ III Platinum^®^ SYBR^®^ Green One-Step qRT-PCR Kit. The quantified genes were the viral RNA codifying to M protein and the cell mRNAs for ZO-1, VCAM, PECAM, TNF- α and MCP-1. Viral RNA was quantitated following the protocol described in Pffafl and colleagues [23], while cell transcripts were analyzed using the protocol described in Schefe and colleagues [24]. In both cases, data were normalized against the β-actin transcript.

### Transmigration assays using J774 monocytes/macrophages

MBECs or MBECs-astrocytes were seeded in Transwells^®^ (Corning; 0.3 cm^2^ with 5-μm pore) as described above. Then, both barrier systems were treated as follows: 1) 15,000 non infected J774 macrophages were put on infected or mock treated MBECs, 2) 15,000 J774 cells infected for 24 h were put on non-infected MBECs, or 3) 15,000 infected J774 cells were seeded on infected MBECs. At 10 h or 24 h after the macrophages were added, the lower chamber medium was withdrawn, and the transmigrated and adhered cells were fixed, counterstained with crystal violet and counted under an inverted microscope (Zeiss Axiovert 40 CFL). The J774 cells were a gift from Dr. Gabriela Delgado at the Laboratory of Immunotoxicology at the Universidad Nacional de Colombia.

### Statistical analysis

Immunofluorescence analyses were performed by counting the cells in eight fields in triplicate in two independent cultures using Image J software. The total cell number and the number of infected cells were used to calculate infection percentages and to perform statistical analyses of angular transformed proportions. For all other experiments, two independent cultures were analyzed in triplicate. Student’s t-tests and Kruskal-Wallis tests were used with Bonferroni *post-hoc* analysis.

## Results

### Obtaining purified cultures of mouse brain endothelial cells and astrocytes

**Fig 1:**
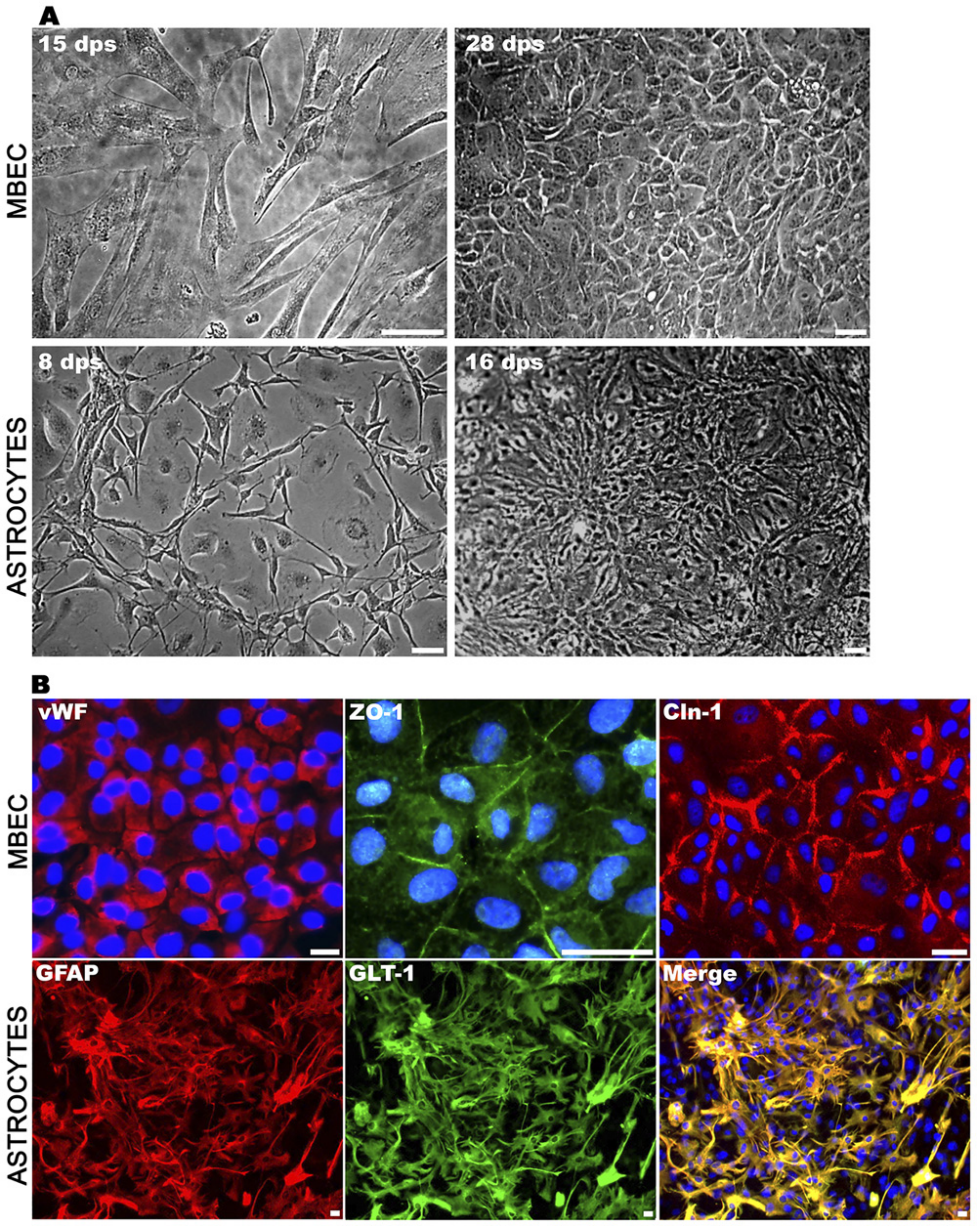
Phase contrast photomicrography of MBECs and astrocytes. **(A)**. Mouse brain endothelial cells were isolated and cultured for 30 days. At 15 days post seeding cells appear fusiform with different length cytoplasmic prolongations. At day 28, cells had polygonal morphology and monolayer acquired the cobblestone endothelial pattern. Bar correspond to 100 μm. To obtain astrocytes, mixed cell brain homogenates were cultured for 14 days and then shaken to detach non-related cells and to purify adhered astrocytes. Figure (left lower) shows the cell morphology at 8 days after purification by shaking, which displayed a typical star-shaped morphology with short cell prolongations. At 16 days post-seeding (right lower panel) cells displayed a morphology with short prolongations like the protoplasmic mature astrocytes. Bar = 20 μm. **(B)**. Immunofluorescence detecting von Willebrand factor (vWF) in 99% of the MBECs. The tight junction proteins ZO-1 and Cln-1 were detected at 28 days post-seeding lining the plasma membrane, confirming the establishment of the barrier. Astrocytes showed 95% purity after staining with the specific markers GFAP and GLT-1. Their morphology was polygonal with many short prolongations and a small nucleus. Bar = 20 μm.

MBECs were obtained from the brain microvasculature of 7-day-old Balb/c mice following the protocol of Weinderfeller and colleagues [18]. The cells initially had spindle-shape morphology, and upon reaching confluence, they acquired a typical non-overlapping cobblestone pattern with 99% purity, according to the expression of the vWF (Fig 1A). The cells exhibited tight junctions, as indicated by the expression of ZO-1 and Cln-1 proteins near the plasma membrane (Fig 1B). Astrocytes were obtained following the methods of Skaper and colleagues [17]. On day 14, the mixed cultures were shaken to purify the cultures. To achieve higher astrocyte enrichment, the cultures were maintained for eight additional days. A population of small cells with cytoplasmic short extensions was observed. After 21 days in culture, this population presented a star-shaped morphology with longer cytoplasmic extensions and had 95% purity, according to the expression of the markers GFAP and GLT-1 (Fig 1A and 1B). The standardized protocols used in these assays allowed us to obtain primary cultures of both MBECs and astrocytes from newborn mice with phenotypic characteristics that were suitable for the establishment of a BBB model [25, 26].

### Endothelial cells, but not astrocytes, are susceptible to DENV infection

**Fig 2:**
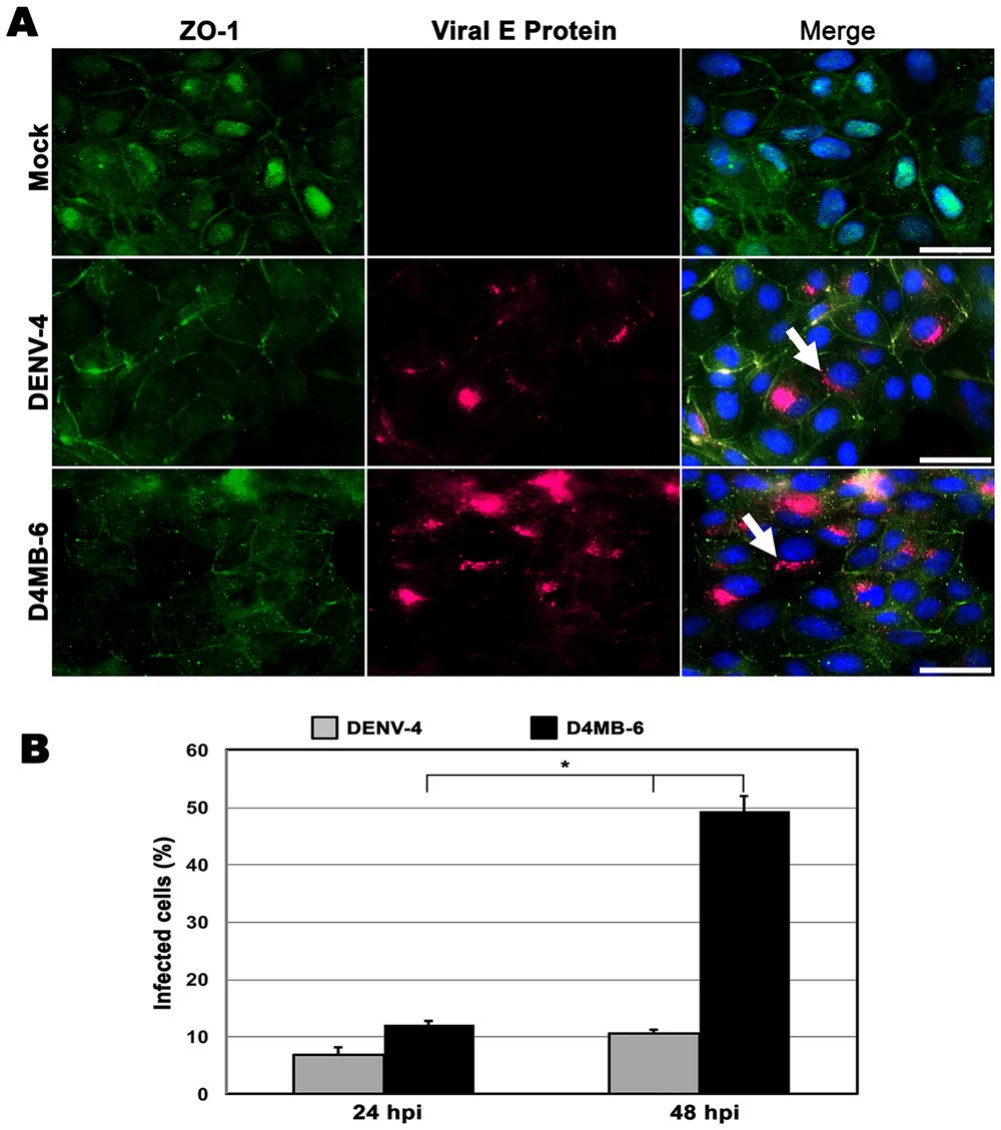
MBEC susceptibility to DENV infection. **(A)**. MBEC cultured on glass coverslips were inoculated for 48 h with mock suspension or infected with DENV-4 or D4MB-6 at an MOI:1 and stained for detecting viral envelope protein (red) and ZO-1 protein (green). Both viruses infected the MBECs and showed perinuclear localization of viral E protein (arrow). On the other hand, the typical plasma membrane localization pattern of ZO-1 was detected in the mock and DENV-4 infected cultures, while in the D4MB-6 infected cells was located mainly in the cytoplasm, in clusters or in a discontinuous pattern in the perimembrane region. Bar = 20 μm. (**B).** Infection percentages of MBEC after 24 or 48 h p.i. Infection proportion was significantly higher with D4MB-6 at 48 h p.i. (49%), with regard to 11% of the cells infected with DENV-4. Data are shown as the mean +/- SD of 3 independent cultures performed by duplicate.

Susceptibility to either the DENV-4 or the neuro-adapted (D4MB-6) variant was assessed using immunofluorescence detection of a viral antigen. The astrocytes were completely refractory to infection, as was previously reported in an in vivo model [9] and by Imbert and colleagues in an in vitro infection model [27]. In contrast, the MBECs were susceptible to infection with both DENV variants, and the NS-1 (S1 Fig) and E proteins (Fig 2A) were found to display a perinuclear pattern. There were differences in the percentages of cells that were infected. At 24 hours post infection (h p.i.), the DENV-4 virus had infected 7% of the MBECs, while 12% of the cells were positive after infection with the D4MB-6 virus. At 48 h p.i., a significant increase was observed in the percentage of infected cells, which corresponded to 11% and 49% of the MBECs that were infected with DENV-4 or D4MB-6, respectively (Fig 2B) (p< 0.05). These results showed that D4MB-6 is more virulent and that MBECs are more susceptible to this strain.

### DENV infection changed the TEER and permeability of BBB models

**Fig 3:**
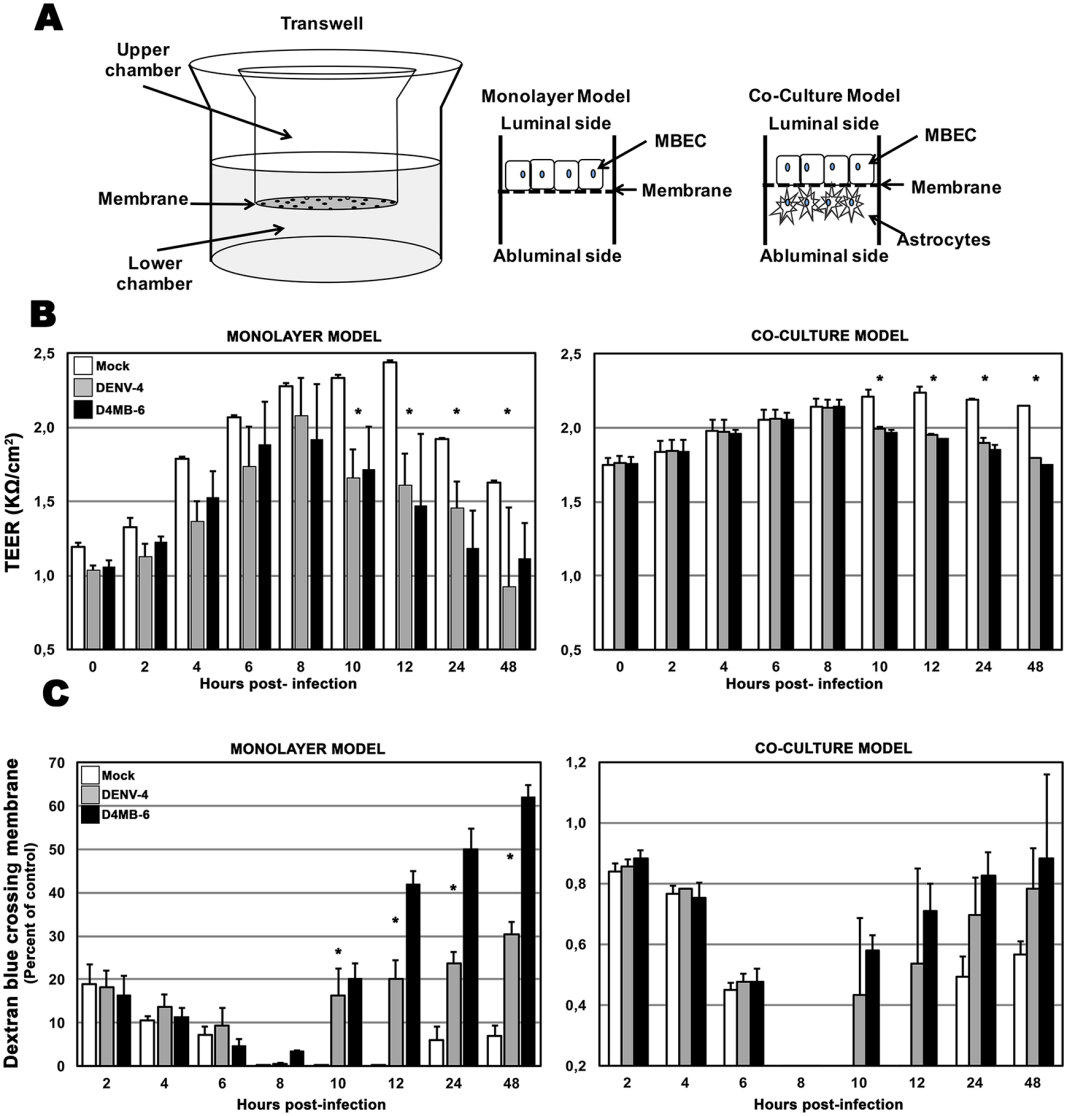
Scheme barrier models and evaluation of TEER and permeability assay in each models. **(A).** Endothelial barrier model scheme. Transwell inserts were used to establish the two barrier models. The first one (Monolayer model) consisted in MBEC cultured on the luminal side of the membrane (upper side) for four days until monolayer reaches TEER values between 1 to 1,5 kΩ. For establishing the second one barrier model (co-culture model), the glial cells were seed on the abluminal side inverting the insert for three days, then it was flipped to the right position before seeding the MBEC in the luminal side. (**B).** Transendothelial electrical resistance. MBEC in each barrier model were infected or treated with mock inoculum. Since 10 h p.i. there was a significant reduction in TEER in DENV-4 and D4MB-6 infected compared with mock-inoculated barrier models. This TEER loss was sustained up to 48 h p.i. (p<0.05, Kruskal-Wallis and Bonferroni tests), however, TEER changes were less drastic in MBEC-astrocytes co-culture with regard to monolayer barrier model. (**C)**. Permeability assay. Using the same culture and infection protocols described above, dextran blue (DB) permeability assays were performed. DB was added in the insert’s upper chamber and at each time point; the lower chamber medium culture was collected to quantify the DB pass through by spectrophotometry. Since 10 to 48 h p.i. the infection with both virus strains induced a significant increase in lower chamber DB concentration coinciding with TEER loss. Barrier damage and DB quantified in the lower medium of MBEC-astrocytes co-culture were too low (between 0,2 to 1,2%) indicating a protective role of astrocytes. Data shown are mean of TEER or DB percentage from triplicates of two independent cultures and SD.

MBECs were seeded on Transwell^®^ cell culture inserts with or without astrocytes (Fig 3A). Four d.p.s. cultures reached a TEER of approximately 1.0–1.5 KΩ and were treated with viruses or control inocula. Different controls were used as follows: i) mock inoculum (non-infected brain lysate), ii) heat-inactivated DENV-4 and D4MB-6 inocula or iii) UV inactivated DENV-4 and D4MB-6 (30 min, 45 watts) (S2 Fig). After infection with each viral strain or treatment with mock or inactivated suspension, the TEER was measured every two hours up to 12 h and at 24 and 48 h.

None of assessed controls showed changes in TEER, even at 48 h p.i. (S3 Fig); therefore, we hereafter report the comparison between infected cells and mock-inoculated cells. In all conditions, TEER values increased up to 8 h, but in DENV-4- and D4MB-6-infected cultures, a significant decrease in TEER values occur compared to the mock group. In this model, MBECs infected with the DENV-4 virus, registered a 29% reduction, while D4MB-6-infected endothelial cells showed a 26.5% reduction in TEER values. This reduction continued until 48 h p.i., when the cultures registered reductions in TEER values of 34% and 44% in DENV-4-infected and D4MB-6-infected cells, respectively (Fig 3B). As expected, the decrease in TEER values at 10 h p.i. was associated with a change in the permeability because 36.7 μg/ml (20%) and 29.8 μg/ml (18%) Blue Dextran was detected in the lower chamber of MBECs infected with DENV-4 or D4MB-6 virus, respectively. The damage to the barrier functions caused by both viruses was irreversible and allowed 54.9 μg/ml (30%) and 110.9 μg/ml (60%) Blue Dextran to pass into the lower chamber of MBECs infected with DENV-4 or D4MB-6, respectively (Fig 3C).

In the co-culture model, high TEER values (2210 Ω) were observed until 8 h p.i. (Fig 3B), and similar to that observed in the monolayer model, a significant decrease in TEER values was observed at 10 h p.i., which corresponded to reductions of 9.5% and 10.8% in the co-cultures infected with the DENV-4 and D4MB-6 virus, respectively. These reductions in TEER values were accompanied by slight changes in the co-culture permeability model, in which 0.83 μg/ml (0.4%) and 0.93 μg/ml (0.6%) Blue Dextran was detected in lower chamber when the cells were infected with DENV-4 or D4MB-6, respectively. At 48 h p.i., these TEER values were reduced by only 18.1% and 16.3%, respectively, and a slight increase in the concentration of Blue Dextran (less than 1%) in the lower chamber was detected in the infected culture system (Fig 3C). These results show that infection with either DENV-4 or neuroadapted variants virus alters barrier function to increase the permeability of the system. However, this damage was attenuated by the presence of astrocytes.

### Infection with DENV induced changes in the subcellular localization of ZO-1 and Cln-1

**Fig 4:**
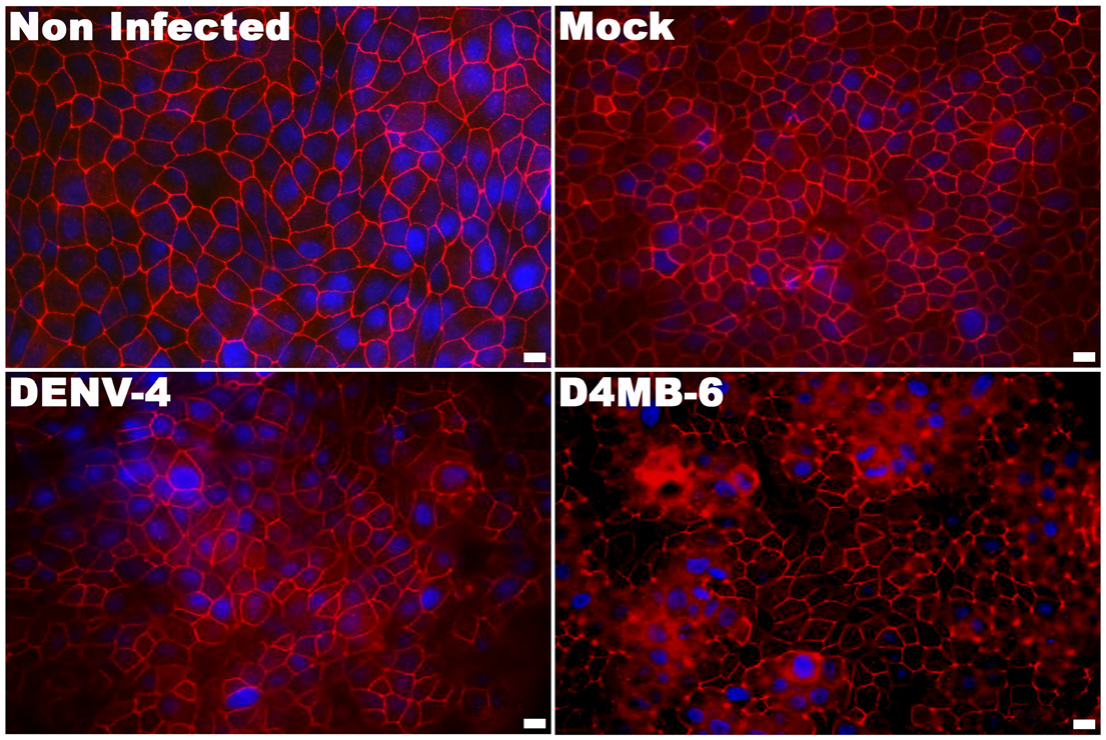
Immunostaining for ZO-1 in MBEC monolayer cultured on the inserts at 10 h p.i. ZO-1 (in red) displayed the expected continuous membrane lining fluorescence pattern along the cell margins of each endothelial cell. At 10 h p.i. with DENV-4 or after mock treatment this fluorescence distribution did not change. By contrast, at 10 h p.i. MBEC infected with D4MB-6 showed slight changes in morphology (some cell detached) and ZO-1 redistribution from margin to cytoplasm, a finding correlated with the initial decrease in TEER values and dextran blue pass through the membrane. Bar = 20 μm.

In addition to evaluating TEER values and permeability, the subcellular localization of ZO-1 was assessed in both monolayer and co-culture models. In mock-inoculated and non-infected cultures, the ZO-1 protein lined the membrane of each cell. After 10 h p.i., D4MB-6 infected MBECs exhibited altered morphology, and ZO-1 showed cytoplasmic localization (Fig 4).

**Fig 5:**
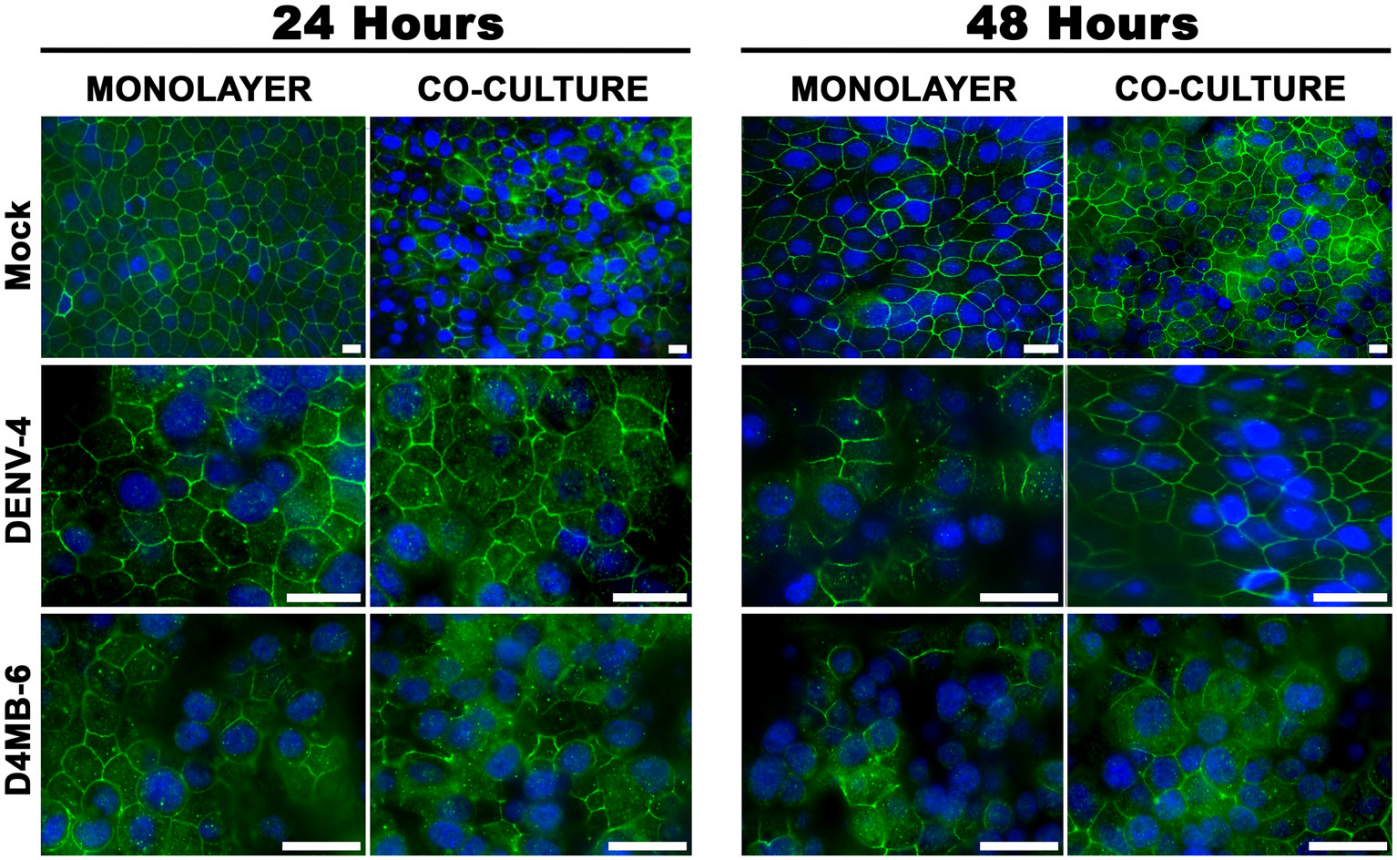
ZO-1 protein redistribution at 24 and 48 h p.i., in DENV infected MBECs. ZO-1 fluorescence pattern (green) in both barrier models infected with DENV-4 and D4MB-6 virus strains were evaluated one, and two days post infection. DENV-4 parental virus infection at 24 h p.i. did not affect the ZO-1 pattern in MBEC cells neither in monolayer barrier model nor in co-culture barrier model. On the contrary, neuroadapted dengue virus induced a ZO-1 re-localization from the cell margin to cytoplasm leaving a disrupted linear fluorescence pattern in the membrane. At 48 h p.i. both viruses induce cell detachment, however in the MBEC monolayer model was more severe with great cell loss and full ZO-1 rearrangement. Bar = 20 μm.

At 24 h p.i., parental DENV-4 infection did not induce changes in the cobblestone appearance in the monolayer or the co-culture models, and most of the cells maintained a cellular distribution of ZO-1 that was similar to that of the controls, although some cells showed cytoplasmic staining. In contrast, at 24 h p.i. in both monolayer and co-culture models, D4MB-6 infection severely affected the ZO-1 distribution pattern, which appeared cytoplasmic and discontinuous in the membrane, but without cell loss evidence. At 48 h p.i., DENV-4 induced a slight loss of cells, and ZO-1 appeared as patches in the cytoplasm and membrane. Meanwhile, the neuroadapted virus induced major changes in ZO-1 localization and cell detachment in the MBEC monolayer system at 48 h p.i. (Fig 5), but these changes did not occur in the MBEC-astrocyte co-culture, which maintained the membrane distribution pattern of ZO-1.

**Fig 6:**
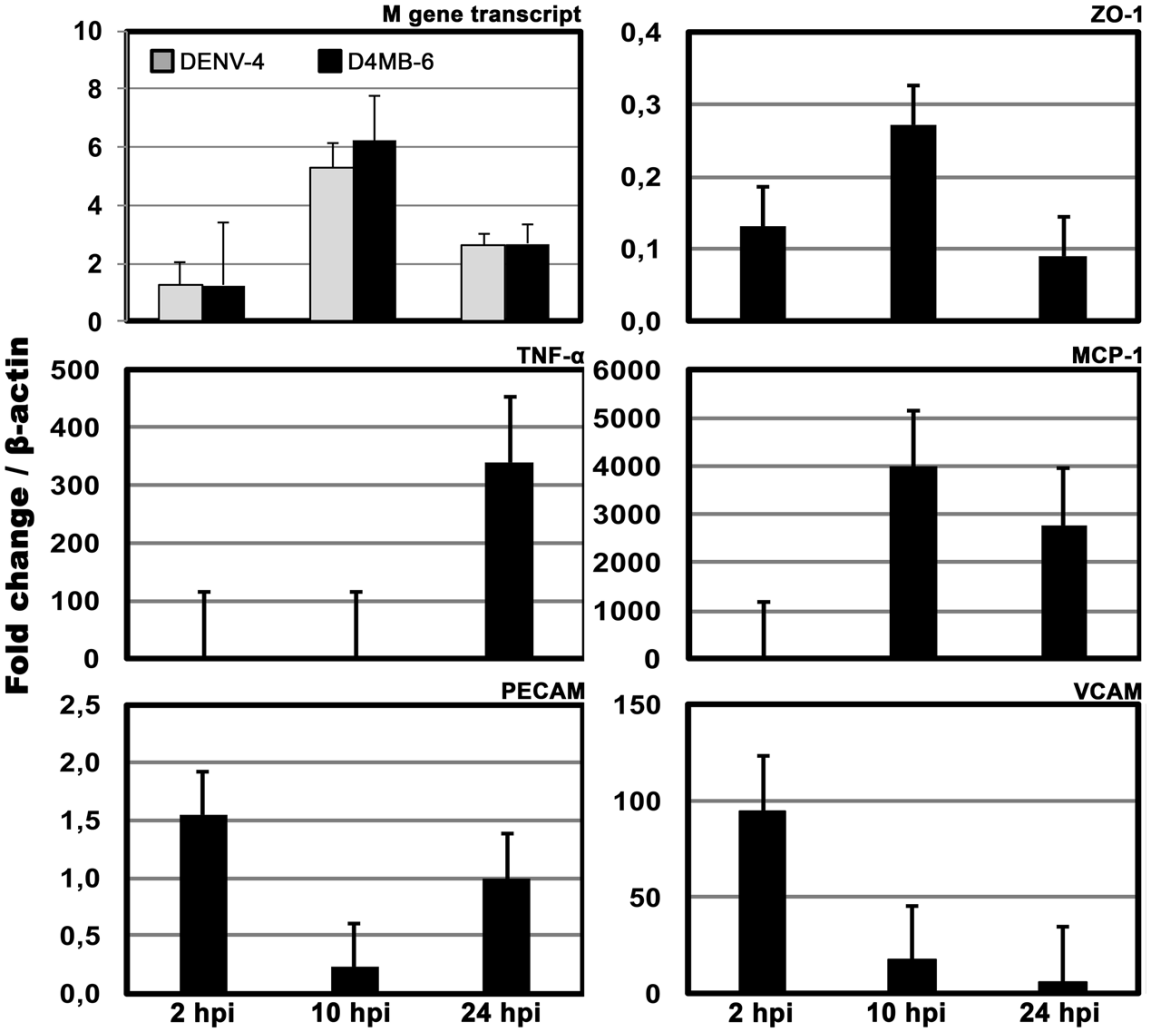
RNA quantitation of viruses, cell proteins, inflammatory mediators and adhesion molecules. MBECs were infected with D4MB-6 and then processed to amplify a segment of dengue M protein gene, and cellular transcripts to ZO-1, TNF-α, MCP-1, PECAM, and VCAM using a quantitative RT-PCR. There were no significant changes in the amount of viral RNA and ZO-1 expression in the evaluated time points. TNF-α transcription was increased at 24 h p.i., while MCP-1 expression was increased at 10 h p.i. PECAM transcription increased at 2 and 24 h p.i. but not at 10 h p.i., while, VCAM expression was increased early (2 h p.i) and then gradually decreased over time. The data are shown as the relative expression obtained from duplicates of two independent experiments. The data were analyzed using the methods described in Pffafl et al [23] or Schefe et al, [24].

Like ZO-1, the cell junction protein Cln-1 changed its localization from the membrane to the cytoplasm, mainly in the MBEC monolayer system after infection with both dengue strains. However, cell damage was more evident after D4MB-6 infection at 48 h p.i., with cells showing cytoplasmic Cln-1 accumulation, with some short lines associated with the membrane, and evident loss of cells (S4 Fig). In spite of these changes in morphology, the expression of the ZO-1 transcript, which was assessed using qPCR, did not change in D4MB-6 infected cells (Fig 6). This finding suggests that MBEC infection induced early reorganization of the actin cytoskeleton or alterations in ZO-1 membrane processing.

### Endothelial barrier damage allows paracellular transport of DENV

To determine the integrity of the barrier and to quantify the passage of free virus across it, the co-culture model was chosen. The virus was titrated in the upper and lower supernatants after MBEC infection. At 2 h p.i., viral particles were not detected in the lower chamber medium or in the cells. At 10 h p.i., we detected approximately 400 PFU/ml of each viral variant in the lower chamber, and at 24 h p.i., there was an increase in the viral titer in both the upper and the lower chambers (Table 1).

**Table 1:** Virus titration in the upper and lower chambers in the co-culture model.

Chamber	hpi	Pfu		Pfu	
**Lower**	2	**DENV-4**	0	**D4MB-6**	0
	10		410		400
	24		2320		3600
	48		4600		4200
**Upper**	2		4000		4000
	10		1600		3000
	12		100		100
	24		10000		10000
	48		16000		13000

Infectious particles were quantitated by plaque assay in the co-culture model, and the values are given in plaque forming units (pfu). At 2 h p.i., the virus was associated with the inoculum. Data are the mean of the replicates of two independent cultures (control) or six replicates of two independent cultures for DENV-infected cultures.

At these same times post-infection, we evaluated the transcription of a segment of viral RNA encoding the M protein and observed the viral RNA associated with the input at 2 h p.i. At 10 and 24 h p.i., we observed a non-significant increase in transcript expression with both viruses (Fig 6). These results indicate that the early steps of infection (entry and viral protein transcription/translation) induced dramatic changes in the barrier functions of MBECs that allowed the paracellular or transcellular transport of the virus from one side of the membrane to the other.

### Infection with D4MB-6 induced endothelial activation and transmigration of monocytes/macrophages

In the MBEC monolayer model, the infection with D4MB-6 induced changes in PECAM and VCAM transcription at 2 h p.i. (1.5 fold and 96 fold, respectively), whereas at 10 h p.i., there was a reduction in the transcription of the same molecules (0.2-fold and 17-fold, respectively), but the MCP-1 messenger was up-regulated (almost 4000-fold). At 24 h p.i., while these transcripts decreased or were no longer detected, TNF-α transcription was up-regulated 340 fold (Fig 6).

Finally, we evaluated immune cell transmigration across infected and non-infected MBECs in both BBB models. Three experimental conditions were evaluated at two time points (see Materials and Methods). A low number of transmigrated immune cells was noted at 24 h in both mock inoculated BBB systems. However, the MBEC monolayer system infected with DENV-4 and D4MB-6 allowed similar J774 transmigration (p>0.05) between the two cell types. Interestingly, endothelial cells infected with D4MB-6 in the MBEC-astrocyte co-culture system allowed significantly greater passage of J774 cells, even when these immune cells were not infected (Table 2) (S5 Fig). These results indicate that DENV infection and endothelial activation contribute to structural BBB damage and favor the transmigration of immune cells, even infected cells.

**Table 2:** Transmigration of J774 macrophages in the barrier models. Mean number of cells (SD).

	**Monolayer Model**								
	**Mock**			**DENV-4**			**D4MB-6**		
	**MBEC**	**J774**	**Trans. Cells**	**MBEC**	**J774**	**Trans. Cells**	**MBEC**	**J774**	**Trans. Cells**
1	**+**	**-**	31 (5)	Inf	No inf	28 (6)	Inf	No inf	42 (10)
2	**-**	**+**	13 (5)	No inf	Inf	28 (8)	No inf	Inf	42 (14)
3	**+**	**+**	25 (8)	Inf	Inf	25 (4)	Inf	Inf	27 (10)
	**Co-culture Model**								
	**Mock**			**DENV-4**			**D4MB-6**		
	**MBEC**	**J774**	**Trans. Cells**	**MBEC**	**J774**	**Trans. Cells**	**MBEC**	**J774**	**Trans. Cells**
1	**+**	-	77 (23)	Inf	No inf	83 (5)	Inf	No inf	74 (27)
2	**-**	+	50 (28)	No inf	Inf	49 (16)	No inf	Inf	45 (17)
3	**+**	+	77 (18)	Inf	Inf	84 (15)	Inf	Inf	75 (22)

Three different experimental conditions were used in both BBB models: 1) Non-infected J774 macrophages were added to mock-inoculated and dengue-infected MBECs. 2) DENV-4- or D4MB-6-infected J774 macrophages were added to mock-inoculated MBECs. 3) J774-infected cells were placed on MBEC-infected monolayers. At 24 h p.i., J774-transmigrated cells (Trans. Cells) were counted in the lower chamber. No differences in J774 cell migration were found in any of the described conditions (1–3) when the MBEC monolayer model was used. J774 cell transmigration was significantly higher when MBECs or both cell types (MBEC and J774) were infected with D4MB-6 in the co-culture model (p = 0.0114, Kruskall-Wallis and Bonferroni post-hoc tests). Data are the mean of three (DENV-4) or six (D4MB-6) independent cultures.

This represents a Trojan horse mechanism of nervous system entry.

## Discussion

The BBB is composed of endothelial cells, pericytes, astrocytes, microglia and neurons, known together as a neurovascular unit (NVU). All of these components are involved in nervous tissue homeostasis. Each cell population responds differently to stimuli and aggression by agents, including viruses. Although neurological manifestations are observed in approximately 5% to 10% of dengue cases, few studies have examined the neuropathogenesis of DENV or its infection of brain tissues [28]. The mechanisms involved in the nervous dysfunction associated with DENV or the pathways used by DENV to enter and spread within nervous tissue are completely unexplored. We previously established a model for infecting the brain in newborn mice using a neuro-adapted DENV-4 strain (D4MB-6), which displays classical neuroinvasiveness and neurotropic features. That virus strain induced changes in the BBB during nervous tissue infection, which suggested that this pathway might be one of the mechanisms used by D4MB-6 to reach nervous tissue [9]. However, there is no information regarding the participation of NVU cells in DENV infection or their contributions to the encephalitis observed in infected animals, but in vitro experiments suggest the active participation of microglial cells, which are differentially activated according the serotype involved [29].

In the present study, we found that MBECs were highly susceptible to infection with DENV-4 and neuro-adapted DENV variants. For the first time, we report that infection directly affects the cellular physiology of mouse brain endothelial cells. Infection and alteration of the endothelium has been described in fatal cases of severe dengue [6, 10, 30]. Avirutnan and colleagues (1998) reported that HUVECs are susceptible to DENV-2 infection and that they secrete immune mediators that are involved in the alteration of vascular permeability, which suggests that infection of the endothelium contributes to dengue pathogenesis by increasing viremia and cytokine levels and by making the endothelium a target for cellular and humoral immune responses [31]. However, a murine model of infection showed that DENV-infected endothelial cells in different organs, including the brain, were associated with bleeding, increased TNF-α production and endothelial barrier dysfunction [32, 33]. The cellular and molecular mechanisms that underlie disruption of the BBB during viral infection are unknown.

In this in vitro model, the compromised integrity and damaged permeability of both the MBEC monolayers and the MBEC-astrocyte co-cultures began at 10 h p.i., when early viral cycle events, such uncoating, transcription and translation, were occurring, which indicated that these steps are sufficient to affect BBB integrity. This is unlike the disruption caused by WNV infection, in which damage and endothelial activation occur after the immune response and cell infiltration, regardless of the infection of NVU cells [32, 34].

Interestingly, we observed that astrocytes are completely refractory to both DENV-4 and the neuro-adapted DENV strains, similar to results observed in mice [9]. Imbert and colleagues, in mouse neuron and astrocyte cultures, also previously reported this astrocyte behavior [27]. In contrast, in fatal cases of dengue infection, viral antigens have been reported in neurons, microglia and astrocytes [28]. These differences may be the result of genomic variations in DENV serotypes that confer changes in cell tropism and the ability of the virus to reach and infect neural and extraneural tissues [35, 36], as has been observed in some pathogenic and non-pathogenic WNV strains [37]. Even when they were not infected, we observed that astrocytes were activated and that they secreted cytokines and chemokines (unpublished results). In the MBEC-astrocyte co-culture model, this may have delayed and reduced the dysfunction of the endothelial barrier after DENV infection (Figs 3 and 5), which indicated that glial cells modulate the MBEC response. These findings are the first to suggest that astrocytes are directly involved in endothelial responses during neuro-infection by DENV. This role is in contrast to what has been previously reported in WNV infection models, in which astrocytes were infected and found to produce proinflammatory mediators and matrix proteinases that degraded the TJP of endothelial cells, which damaged the barrier [38]. Similarly, Chen and colleagues demonstrated that JEV disrupted the BBB, partially via the infection and activation of pericytes, which secreted IL-6 and induced the activation of the ubiquitin proteasome system and the subsequent degradation of the protein ZO-1 [39]. Therefore, these data show that the NVU cells contribute to the onset and progression of BBB disruption in different ways, but in the particular case of DENV, astrocytes play a protective role that stabilizes the barrier.

We found that DENV infection caused the loss of the cobblestone appearance of MBECs and alterations in cell contours and the perimeter, area and localization of ZO-1 and Cln-1 protein, which suggested that DENV infection was able to alter the cytoskeleton, the organization and assembly of the TJP and the integrity and permeability of the BBB. DENV infection induces cytoskeletal reorganization, as was reported in EA.hy926 endothelial cells, in which the expression and localization of actin were changed to modify the cytoarchitecture and integrity of the monolayer [40]. Similarly, using dermal microvascular endothelial cells (HMEC-1), Talavera and colleagues found that DENV infection and replication induced alterations in permeability that were associated with actin filaments and occludin relocalization [41]. Despite these findings, it is clear that the BBB alterations induced by DENV infection allowed different methods for the virus to reach the nervous stroma. It has been reported that the virus can be transported via a paracellular route or via transcytosis. We observed that the virus passed through the membrane early (at 10 h p.i.) when changes in permeability and TEER values were observed, a finding compatible with paracellular transport.

Paracellular transport depends on changes in cell morphology that occur after substantial alterations to tight junctions and on activation of endothelial cells. Here, both the MBEC monolayer and the MBEC-astrocyte co-culture exhibited initially high TEER values that indicated close connections mediated by TJP. However, at 10 h p.i., a significant reduction in TEER values was observed, which indicated an increase in permeability and suggested disruption in the integrity of the barrier that was associated with changes in TJP localization. Other researchers have reported BBB alterations that are accompanied by changes in TJP transcription, translation, degradation, phosphorylation and subcellular distribution, which combine to alter the permeability of the barrier [4]. We did not observe changes in transcription of ZO-1, which suggested that infection might alter membrane localization of proteins by changing phosphorylation patterns [42]. In contrast, Kanlaya and colleagues [40] observed a decrease in ZO-1 expression in an endothelial cell line. Infection by neuro-adapted D4MB-6 virus may therefore induce alterations in the phosphorylation or degradation of ZO-1 as a viral strategy to disrupt BBB integrity and promote entry. The observation of the early re-localization of ZO-1 and Cln-1 may explain how viruses pass through the endothelial monolayer in a paracellular manner. Agrawal and colleagues reported that JEV infection and replication in epithelial and endothelial cells resulted in the late opening of TJPs (at 48 h p.i.) without the presence of pro-inflammatory agents [43]. For WNV, endothelial passage was observed only at later times; therefore, it was assumed that the entry of WNV into the CNS is primarily mediated by transcellular transport [44].

Transcytosis was previously reported in a DENV infection model [45, 46, 47] and in other flaviviruses, such as WNV [44, 48]. In this work, we show that MBEC activation induced by the D4MB-6 neuro-adapted virus favored the passage of monocytes from the luminal to the abluminal side. Infection with either DENV-4 or the neuro-adapted strain changed the MBEC phenotype to one with a pro-inflammatory status that promoted expression of cytokines and chemokines, in addition to other inflammatory molecules that promote leukocyte adherence and recruitment [11]. In particular, we observed that endothelial activation, which occurred as early as 2 h p.i. in D4MB-6-infected cells, was associated with progressive and coordinated changes in the expression of VCAM, PECAM, TNF-α and MCP-1. These molecules are strongly involved in endothelial remodeling and BBB disruption. For example, at 24 h p.i., we observed that transmigration of J774 cells coincided with expression of MCP-1, PECAM and TNF-α, which are molecules known to promote attraction and migration of immune cells and opening of the TJP [34, 49, 50, 51, 52]. DENV infection is known to induce endothelial cell activation and production of pro-inflammatory cytokines, adhesion molecules and other agents, such as nitric oxide [53], similar to what was observed in brain microvasculature endothelial cells. In conclusion, endothelial morphological changes, in combination with changes in tight junctions, are different sides of the same issue and may be strategies used by DENV at different time points to foster the entry and spread of viral particles.

**Fig 7:**
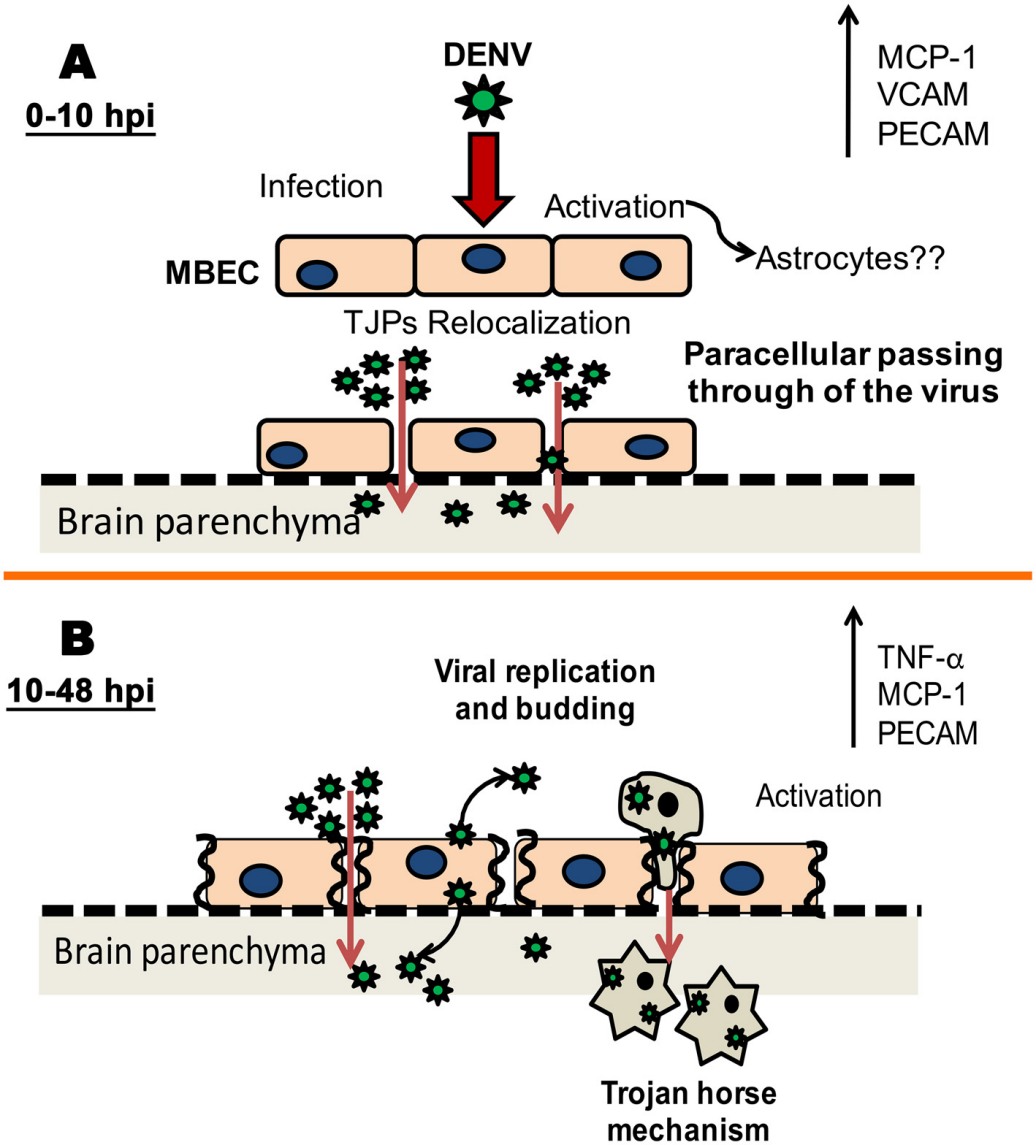
DENV transmigration model of passage through the BBB. **(A).** After the virus inoculation, MBECs are activated and/or infected, inducing early TJP relocalization and the expression of MCP-1 and adhesion molecules (10 h p.i.). These changes allowed viral paracellular transport by which the particles passed through to abluminal side. Adhesion molecule expression also changed, bringing immune cells closer and favor rolling. (**B).** After viral replication in MBEC, transcellular virus transport occurs, and the integrity of the barrier is damaged, resulting in an increase in virus paracellular transport to the abluminal side. MBEC activation and the production of inflammatory mediators promote leukocyte adhesion and transmigration, which carries the virus through via a Trojan horse mechanism.

Finally, our findings suggest that the entry and spread of DENV in nervous tissue may be explained by (i) transmigration of leukocytes and (ii) paracellular transport of DENV through the cerebrovascular endothelium. Here, MBECs infected with either DENV-4 or neuro-adapted DENV strains showed gradual alterations in the integrity of the BBB. The early steps in the viral cycle that occurred during the first 10 h p.i. were sufficient to induce changes in the structure of the barrier and to up-regulate inflammatory mediators. These changes allowed the passage of free viruses from the luminal to the abluminal side of the membrane (Fig 7A). Infection and efficient replication of the virus in MBECs induced progression in the damage of the endothelial monolayer and TNF- α and MCP-1 production, which favored leukocyte transmigration. We therefore suggest that a Trojan horse mechanism is used at a late time point and that this phenomenon, as well as viral paracellular transport, is the main strategy used by DENV to reach the nervous system (Fig 7B).

## Supporting Information

S1 Fig Non-structural 1 (NS-1) protein detection in infected MBEC. Dengue NS-1 protein immunofluorescence (red) and ZO-1 (green) detection at 48 h p.i. Viral antigen appears at perinuclear region in some cultured cells and ZO-1 had the typical marginal pattern in all the endothelial cells. Bar: 20 μm
(TIF)

S2 Fig TEER dynamics analyzed in both barrier models. A gradual increase in TEER values occurs during the first days after start the culture until reach resistances between 1 to 1,5 KΩ, after there were variations associated with culture dynamics (cell loss, migration, proliferation). TEER values were significantly higher in the co-culture model. DENV infections were performed starting on the fourth day post-seeding.
(TIF)

S3 Fig TEER and permeability assay in control cultures. **A.** MBEC cultured as described in Material and Methods, were treated with different kinds of control inocula such as Mock inoculum (non-infected brain lysate), D4MB-6 virus inactivated at 56°C for 1 h, D4MB-6 UV-inactivated (30 min at 45 watts). Treatment with these control inocula did not induce changes in electrical resistance. **B.** Permeability assay using Dextran Blue (DB) added to the upper chamber and subsequent quantitation in lower chamber by spectrophotometry. In the same way, there were not significant changes in either TEER or DB pass through in any of treatments or evaluated time points. Data are shown as the TEER mean or dextran blue percentage from triplicates of two independent cultures and the corresponding standard deviations.
(TIF)

S4 Fig Claudin-1 redistribution in DENV infected MBECs. Evident changes of Cln-1 localization since 24 h p.i. using both DENV-4 and D4MB-6 virus to infect MBEC. Neuroadapted virus induced a higher Cln-1 cytoplasmic accumulation, although there was also discontinuous marginal staining in some cells. These changes are more relevant the MBEC monolayer barrier system when the D4MB-6 was used. At 48 h p.i., cell loss and Cln-1 re-localization were complete. Bar = 20 μm
(TIF)

S5 Fig J774 cells transmigrated in both models. (XLSX)

## Abbreviations

BBB: Blood brain-barrier
bFGF: Basic fibroblast growth factor
BSA: Bovine serum albumin
Cln-1: Claudin-1
CNS: Central nervous system
DENV: Dengue virus
DENV-4: Dengue virus serotype 4
D4MB-6: Neuroadapted DENV strain
GFAP: Glial fibrillary acidic protein
GLT-1: Glutamate transporter
H P.I: Hours post-infection
JEV: Japanese encephalitis virus
MM: Maintenance medium
MBECs: Mouse brain endothelial cells
MOCK: Brain lysate
MOI: Multiplicity of infection
NVU: Neurovascular Unit
PFA: Paraformaldehyde
PFU: Plaque forming units
P.I: Post-infection
TEER: Transendothelial electrical resistance
vWF: von Willebrand Factor
WNV: West Nile Virus
