# Supplementary figures and images for: In Vitro Infection with Dengue Virus Induces Changes in the Structure and Function of the Mouse Brain Endothelium
Source: PLoS One. 2016 Jun 23;11(6):e0157786. doi: 10.1371/journal.pone.0157786 (PMC4919088; doi:10.1371/journal.pone.0157786)

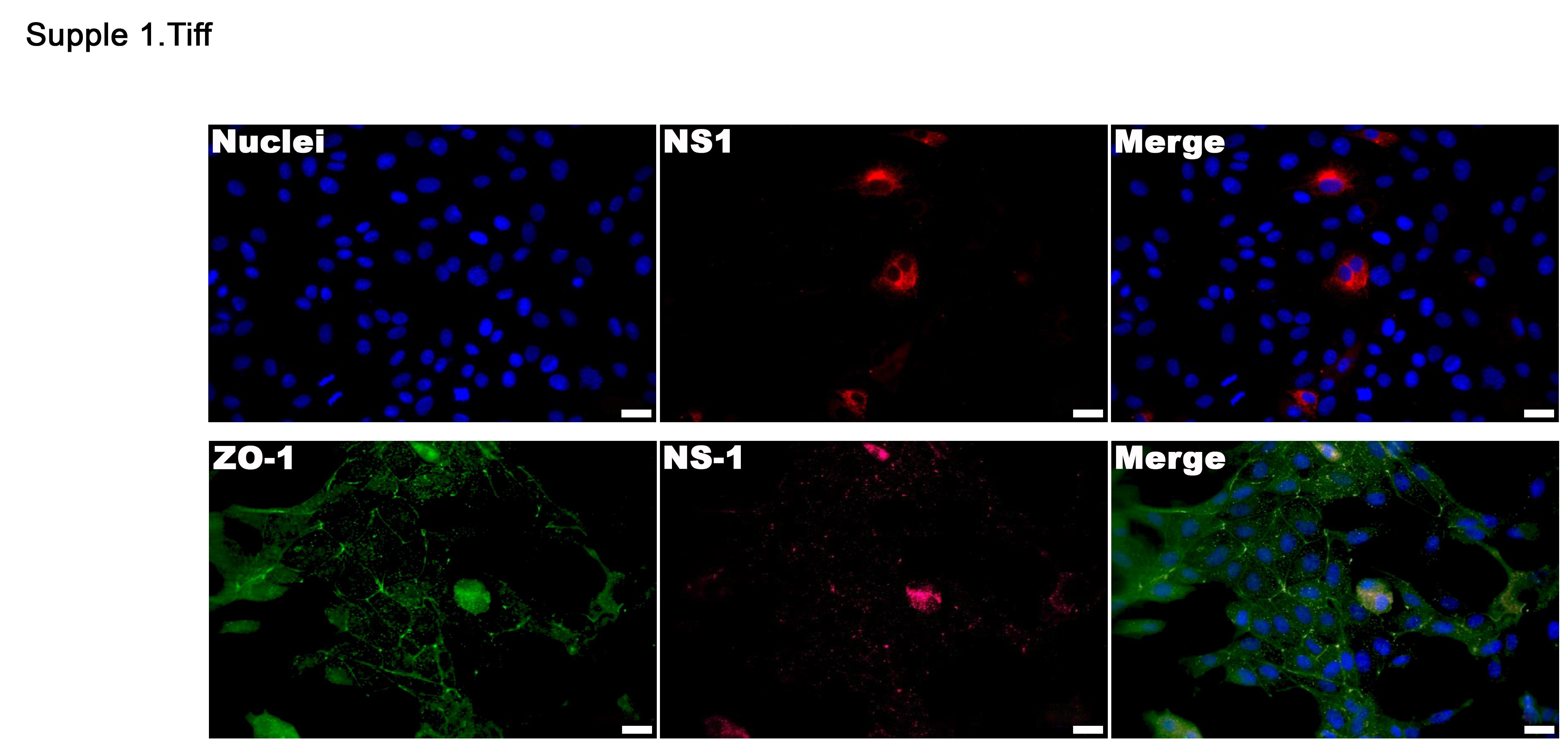

Supplement: S1 Fig — Dengue NS-1 protein immunofluorescence (red) and ZO-1 (green) detection at 48 h p.i. Viral antigen appears at perinuclear region in some cultured cells and ZO-1 had the typical marginal pattern in all the endothelial cells. Bar: 20 μm (TIF) [file pone.0157786.s001.tif]

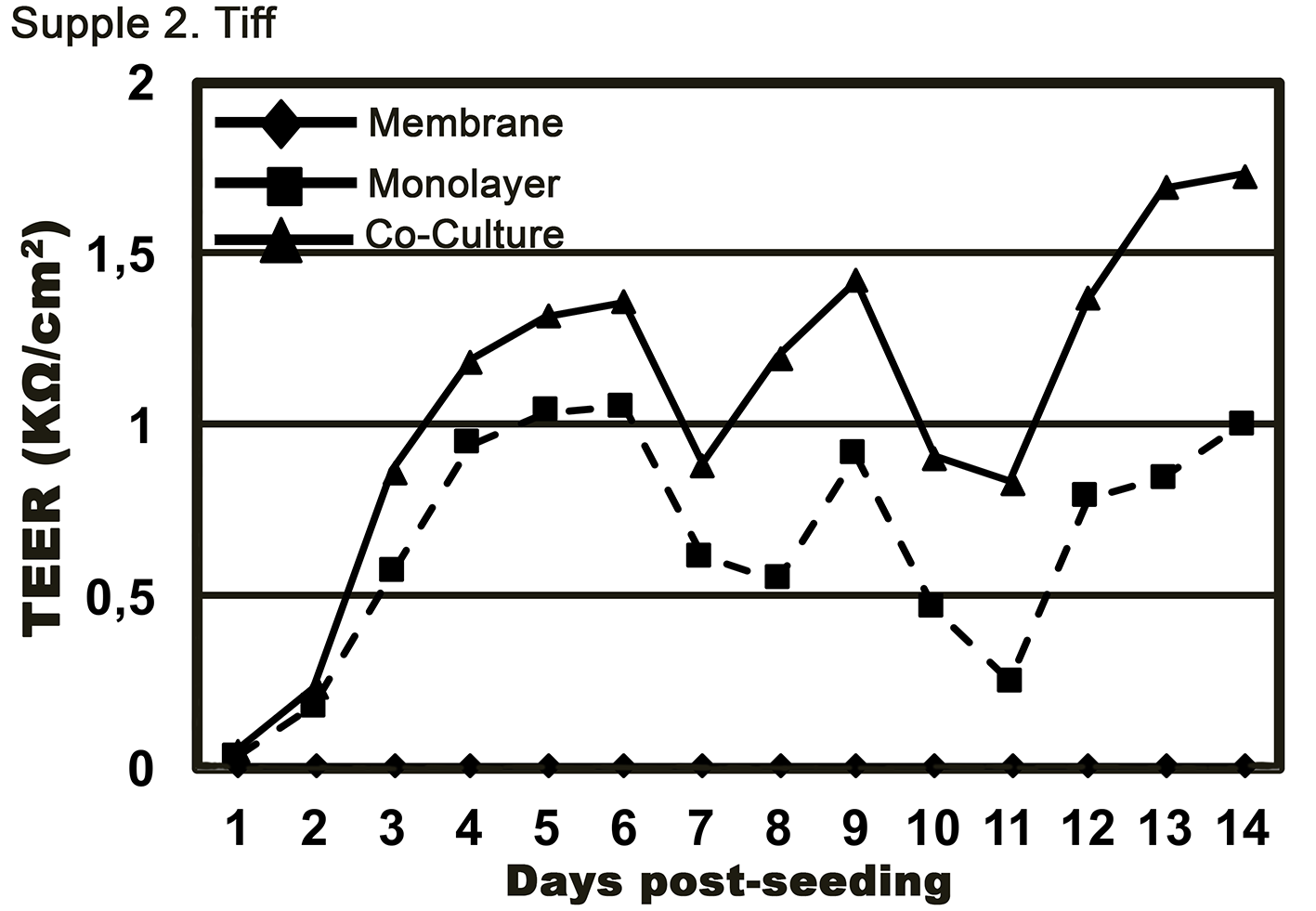

Supplement: S2 Fig — A gradual increase in TEER values occurs during the first days after start the culture until reach resistances between 1 to 1,5 KΩ, after there were variations associated with culture dynamics (cell loss, migration, proliferation). TEER values were significantly higher in the co-culture model. DENV infections were performed starting on the fourth day post-seeding. (TIF) [file pone.0157786.s002.tif]

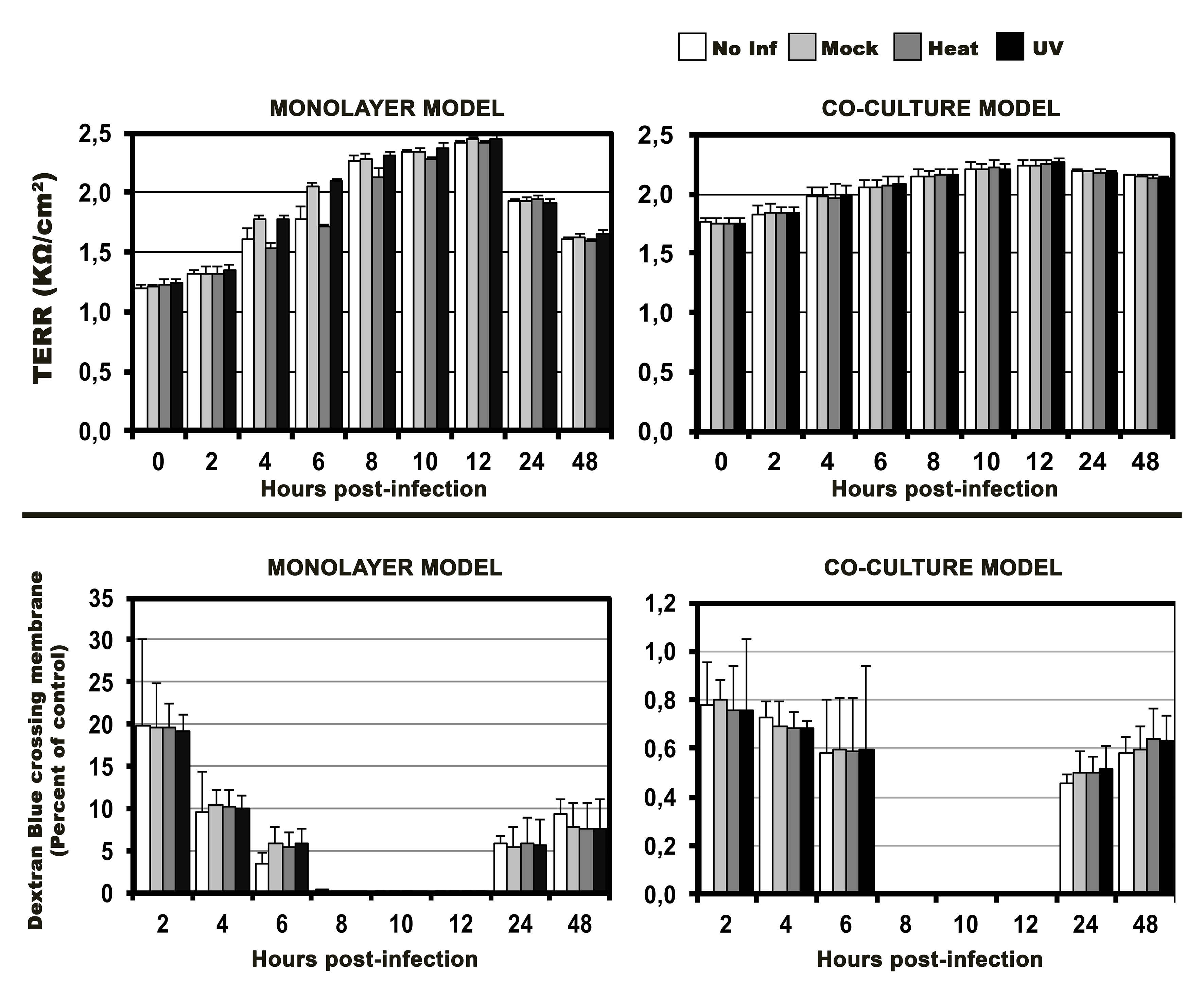

Supplement: S3 Fig — A. MBEC cultured as described in Material and Methods, were treated with different kinds of control inocula such as Mock inoculum (non-infected brain lysate), D4MB-6 virus inactivated at 56°C for 1 h, D4MB-6 UV-inactivated (30 min at 45 watts). Treatment with these control inocula did not induce changes in electrical resistance. B. Permeability assay using Dextran Blue (DB) added to the upper chamber and subsequent quantitation in lower chamber by spectrophotometry. In the same way, there were not significant changes in either TEER or DB pass through in any of treatments or evaluated time points. Data are shown as the TEER mean or dextran blue percentage from triplicates of two independent cultures and the corresponding standard deviations. (TIF) [file pone.0157786.s003.tif]

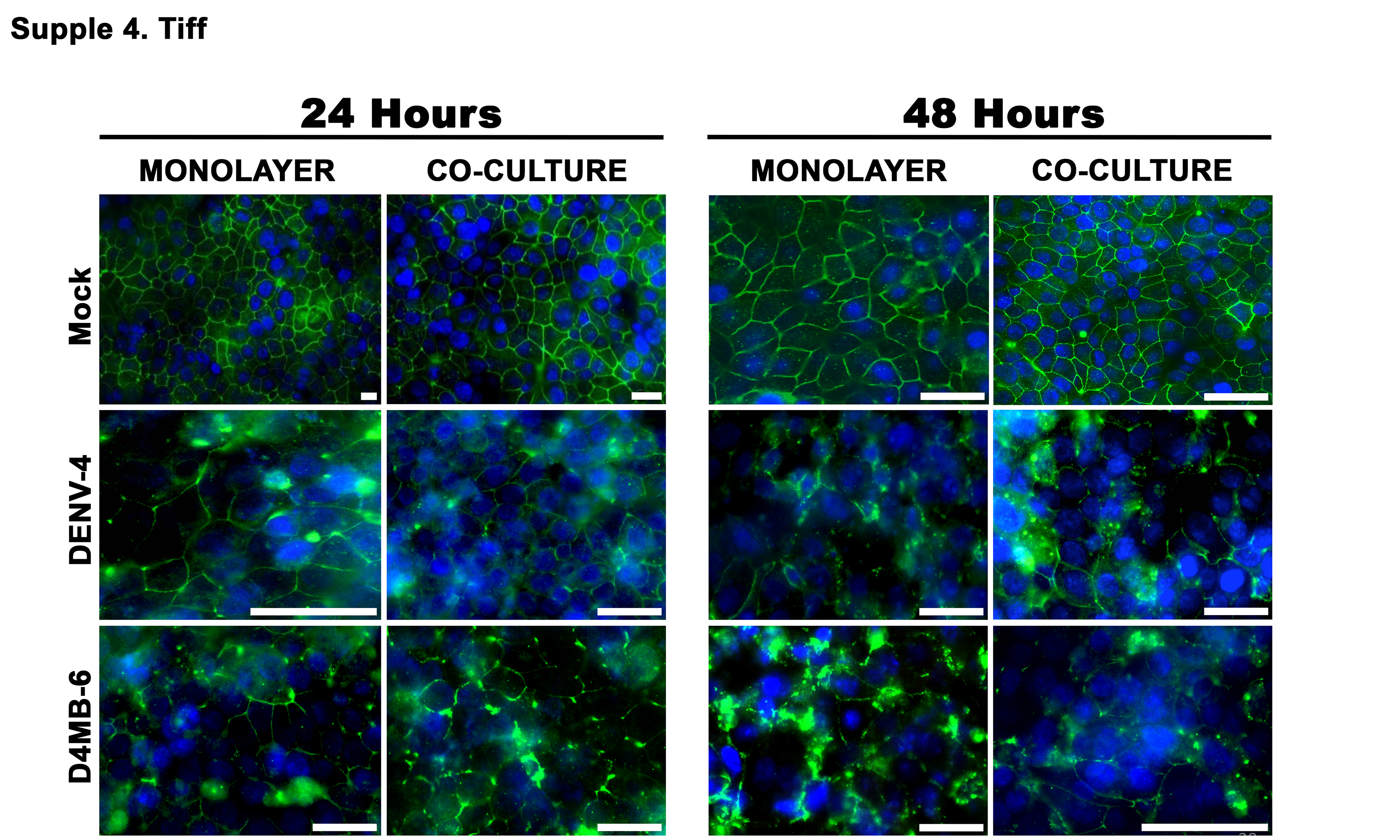

Supplement: S4 Fig — Evident changes of Cln-1 localization since 24 h p.i. using both DENV-4 and D4MB-6 virus to infect MBEC. Neuroadapted virus induced a higher Cln-1 cytoplasmic accumulation, although there was also discontinuous marginal staining in some cells. These changes are more relevant the MBEC monolayer barrier system when the D4MB-6 was used. At 48 h p.i., cell loss and Cln-1 re-localization were complete. Bar = 20 μm (TIF) [file pone.0157786.s004.tif]
